# Investigating prenatal and perinatal factors on meconium microbiota: a systematic review and cohort study

**DOI:** 10.1038/s41390-023-02783-z

**Published:** 2023-08-17

**Authors:** Jenni Turunen, Mysore V. Tejesvi, Niko Paalanne, Tytti Pokka, Sajeen Bahadur Amatya, Surbhi Mishra, Anna Kaisanlahti, Justus Reunanen, Terhi Tapiainen

**Affiliations:** 1 Research Unit of Clinical Medicine, University of Oulu, Oulu, Finland; 2 Biocenter Oulu, University of Oulu, Oulu, Finland; 3 Ecology and Genetics, Faculty of Science, University of Oulu, Oulu, Finland; 4 Department of Pediatrics and Adolescent Medicine, Oulu University Hospital, Oulu, Finland; 5 Research Service Unit, Oulu University Hospital, Oulu, Finland; 6 Research Unit of Translational Medicine, University of Oulu, Oulu, Finland

## Abstract

**Background:**

The first-pass meconium has been suggested as a proxy for the fetal gut microbiota because it is formed *in utero*. This systematic review and cohort study investigated how pre- and perinatal factors influence the composition of the meconium microbiota.

**Methods:**

We performed the systematic review using Covidence by searching PubMed, Scopus, and Web of Science databases with the search terms “meconium microbiome” and “meconium microbiota”. In the cohort study, we performed 16 S rRNA gene sequencing on 393 meconium samples and analyzed the sequencing data using QIIME2.

**Results:**

Our systematic review identified 69 studies exploring prenatal factors, immediate perinatal factors, and microbial composition in relation to subsequent health of infants but gave only limited comparative evidence regarding factors related to the composition of the meconium microbiota. The cohort study pointed to a low-biomass microbiota consisting of the phyla Firmicutes, Proteobacteria and Actinobacteriota and the genera *Staphylococcus*, *Escherichia-Shigella* and *Lactobacillus*, and indicated that immediate perinatal factors affected the composition of the meconium microbiota more than did prenatal factors.

**Conclusions:**

This finding supports the idea that the meconium microbiota mostly starts developing during delivery.

**Impact:**

- It is unclear when the first-pass meconium microbiota develops, and what are the sources of the colonization.
- In this systematic review, we found 69 studies exploring prenatal factors, immediate perinatal factors, and microbial composition relative to subsequent health of infants, but there was no consensus on the factors affecting the meconium microbiota development.
- In this cohort study, immediate perinatal factors markedly affected the meconium microbiota development while prenatal factors had little effect on it.
- As the meconium microbiota composition was influenced by immediate perinatal factors, the present study supports the idea that the initial gut microbiota develops mainly during delivery.

## Introduction

The human gut microbiota has become a topic of interest due to its health effects, and the first-pass meconium, i.e., the first stool after birth, formed *in utero*, is the first easily available sample for investigating the development of this gut microbiota in newborn infants. The meconium microbiota has previously been characterized in several studies,^1–8^ and interestingly, its composition has been found to be associated with the subsequent health of the children, including infantile colic and overweight.^9–11^

The microbiota of the first-pass meconium has been suggested as a possible proxy for the fetal gut microbiota^12,13^ because it is formed in the gut before birth. Alternatively, the initial colonization may start perinatally during delivery.^6,14–16^ As the timing of the initial colonization of the gut is still unclear, we hypothesized that the influence of prenatal and immediate perinatal factors, including exposure to antibiotics at birth and the mode of delivery, should be compared to elucidate the timing of the early colonization process. Furthermore, as meconium samples are prone to contamination because of their low biomass, there is limited evidence regarding their microbial communities from studies maintaining rigorous control over contamination.^17–21^

This paper presents a systematic review of previous investigations into the microbiota of the first-pass meconium, followed by a comparison of the effects of prenatal and immediate perinatal factors on the microbiota composition of the first stool in a cohort study of 393 newborn infants using a robust means of controlling for environmental contaminants.

## Materials and methods

### Systematic review of the literature

We performed a systematic review of the literature on the meconium microbiota using Covidence, a web-based collaboration software platform that streamlines the production of systematic and other literature reviews (Covidence systematic review software, Veritas Health Innovation, Melbourne, Australia. Available at www.covidence.org). We searched PubMed (date: 1.12.2022), Scopus (date: 1.12.2022), and Web of Science (2.12.2022) and used terms “meconium microbiome” and “meconium microbiota” for the literature search. The results were filtered to include only reports written in English and based on original research employing human samples. The search details can be seen in Supplementary Information 1. In the Covidence platform we removed duplicates and performed title and abstract screening, and separately full text screening, during which we also removed articles that did not study meconium or did not include samples of human origin and the results of 16 S sequencing analysis. After evaluating the selected references, we created a summary table with the following information regarding the publications: title, digital object identifier (DOI), number of meconium samples, prenatal factors (maternal characteristics, environmental factors during pregnancy), immediate perinatal factors (exposure to antibiotics during birth, delivery mode), other factors (newborn characteristics and health outcomes), additional methods beyond standard 16 S sequencing such as whole-genome shotgun sequencing and metabolomic analyses, the use of metabolic pathway prediction tools in the analysis, and reported use of negative and positive controls during the laboratory work. Finally, we created a flowchart of the review process using PRISMA.^22^

### Study design and supervision

For the cohort study, we recruited mothers and their newborn infants treated in Oulu University Hospital, Oulu, Finland, between April 27th, 2016, and December 19th, 2018. The hospital serves as the sole primary delivery hospital for a population of 410,000 people, with about 4000 annual births. We invited all mothers delivering via Caesarean section during that period to participate. The pregnant women were recruited by the nurse at their preoperative appointment, and for each participant giving birth via C-section we recruited one mother with a vaginal delivery at the same time. All the participants gave their written informed consent. The Ethics Committee of the Central Finland Hospital District, Finland, found the study plan ethically acceptable (EETTMK:3/2016).

### Study population

Altogether 508 mother-neonate pairs were enrolled during the recruitment period: 253 mothers in the vaginal birth group and 255 in the Caesarian section group. For 11 mothers the mode of delivery was changed from vaginal to C-section after recruitment, so that eventually, 242 neonates were born vaginally and 266 via C-section. A subset of these cases has been used in previous reports.^6,23^ Thus, we had 393 meconium samples with enough fecal material for the present study. In addition, 50 negative control samples of sterile water (HyClone™ HyPure, Thermo Fisher Scientific) were processed with the meconium samples to control for environmental contamination.

### Collection of first-pass meconium samples

The first-pass meconium samples were collected as soon as possible after the birth from the diaper to collection tubes using a plastic spoon in the maternity ward and stored at –80 °C until analyses. During analyses, samples were stored at –20 °C.

### DNA extraction

DNA was extracted using the DNeasy PowerSoil Pro kit (Qiagen, Hilden, Germany), by suspending 200 mg of sample in 1 ml of PBS and performing bead beating homogenization using Tissuelyzer at 25 Hz for 2 min. Tissuelyzer is recommended to samples which are hard to suspend in the extraction buffer, such as meconium samples, thus, we replaced the kit’s Vortex Adapter instructions with it in the case of meconium samples with more raw material. The samples were then left to incubate on ice for 1 min and homogenization was repeated 1–3 times. After the homogenization, the manufacturer’s instructions were followed. The negative controls and meconium samples with little material were homogenized using the kit’s Vortex Adapter instructions. We performed the extractions on a QIAcube DNA extraction machine (Qiagen). The final elution volume was set at 100 µl, and the quantity and purity of the DNA were measured using a Nanodrop Spectrophotometer (Thermo Scientific).

### PCR, sequencing and sequence preprocessing

PCR and sequencing of the V3-V4 hypervariable region of the bacterial 16 S gene took place in the DNA Sequencing and Genomics Laboratory, Institute of Biotechnology, University of Helsinki. PCR was performed using the Phusion HotStart enzyme and a 2-step protocol that started with a PCR using a mix of 341 F (5′ CCTACGGGNGGCWGCAG 3′) and 785 R (5′ GACTACHVGGGTATCTAATCC 3′) primers that included truncated overhangs for the Illumina TruSeq adapter (Supplementary Information 2). For the second PCR dual-index primers selected using Barcosel^24^ were used to target the overhangs in the first PCR. The ZymoBIOMICS™ Microbial Community DNA Standard was used as a mock community, and the PCR products were pooled, purified and sequenced using a 600 cycle v3 sequencing kit on MiSeq (Illumina, San Diego, CA).

The analysis was conducted with QIIME2 (versions 2021.2 and 2022.8).^25^ The reads were imported in paired end phred 33 format and demultiplexed. Denoising and chimera filtering were performed with DADA2,^26^ and the reads were trimmed at base 15 and truncated at base 280 for forward reads and at 220 for reverse reads. Environmental contamination in the 50 negative controls was identified using the R package decontam (version 1.16.0).^27^ Contamination from the meconium samples was removed by a prevalence-based method where the presence and absence of each sequence is compared between negative controls and true samples. The threshold was set to 0.5, meaning that the sequence is considered a contaminant if it is more prevalent in negative controls than in true samples. Taxa identified as Mitochondria, Eukaryota, Cyanobacteria, and Archaea were omitted from the analyses.^6^ Overall, 23,481,047 reads were left for further analysis. Alpha rarefaction plots were examined to choose a sampling depth, and the samples were rarefied at 31,069, excluding reads with lower read counts from diversity analyses.

### Analysis

To analyze the effect of delivery mode and exposure to intrapartum antibiotics, we grouped the meconium samples into vaginal delivery group without intrapartum antibiotic treatment, vaginal delivery group with intrapartum antibiotic treatment and C-section delivery group. C-section delivery samples were not grouped based on intrapartum antibiotic exposure due to nearly all C-sectionally born infants being exposed to antibiotics. For the within-sample diversity, known as alpha diversity, we calculated Shannon Index and observed features which are unique DNA sequences down to a single nucleotide, and therefore more precise than the operational taxonomic units (OTUs) commonly used previously, to measure differences between the sample types. Statistical significance was confirmed using the Kruskal–Wallis *H* test with Benjamini–Hochberg correction for p-values to control the false discovery rate (FDR) for multiple comparisons. To calculate between-sample diversity, or beta diversity, we performed Principal Coordinate Analysis using Bray–Curtis Dissimilarity. Statistical significance was confirmed using PERMANOVA with a threshold of *p* < 0.05. The relative abundances in the taxonomy were calculated at the phylum and genus levels using SILVA database (version 138)^28^ and statistical significances of these abundances were evaulated using analysis of composition of microbiomes (ANCOM).^29^ In ANCOM analysis, pairwise tests are performed on the abundance of each feature between sample groups, and an automatic threshold by the framework is set to the number of rejected null hypothesis results known as W. If the value of W exceeds the threshold, the feature is considered differentially abundant between the sample groups. In the case of the most interesting phyla and genera we performed multivariate regression analysis using a linear mixed model, including several prenatal and perinatal factors simultaneously. We used Phylogenetic Investigation of Communities by Reconstruction of Unobserved States (PICRUSt2) (version 2.5.1)^30^ to analyze the predicted metabolic pathways by aligning amplicon sequence variants (ASVs) produced during the denoising phase to a reference tree, using HMMER (hmmer.org) for alignment and SEPP^31^ for placements in the tree, with Integrated Microbial Genomes (IMG) database.^32^ The resulting tree file was produced with GAPPA.^33^ The hidden-state prediction of gene families was performed using a script based on the castor R package.^34^ The pathway abundance predictions were made by regrouping Enzyme Classification (EC) numbers into MetaCyc reactions and predicting the metabolic pathways using MinPath.^35^ Statistical significances of the metabolic pathway abundances were confirmed with ANCOM. Finally, we used machine learning to create a learning classifier that can predict metadata in our samples and identify the most important genera for the predictions. The random forest nested cross-validation (NCV) learning method was used, and the number of estimators was set at 200. The figures were drawn using ggplot2 (version 3.3.6), grid (version 4.2.1) and GridExtra (version 2.3).

## Results

### Systematic review of the literature

**Fig. 1:**
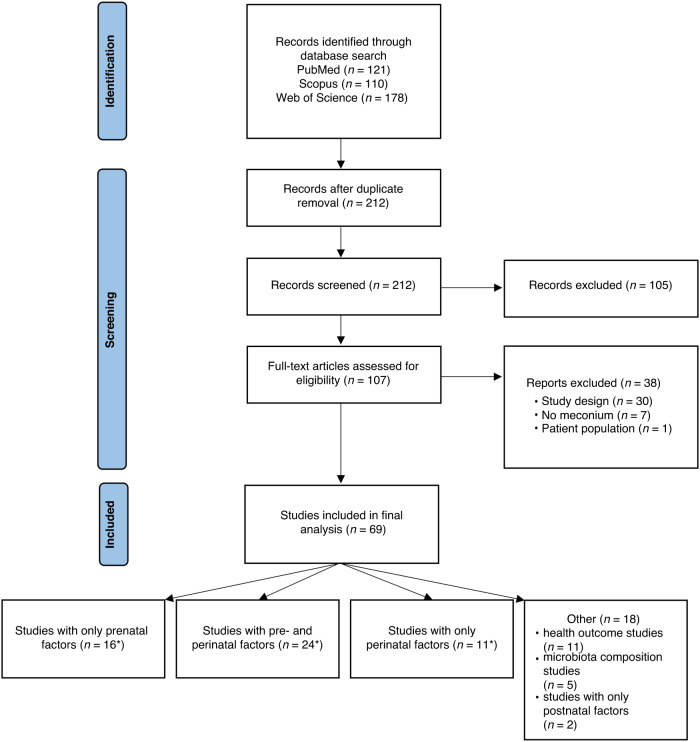
Flowchart of the systematic review process (drawn using PRISMA). *Also includes postnatal factors.

The search through the databases yielded 409 publications, 197 of which were removed as duplicates. Altogether 212 publications were screened by their titles and abstracts (Fig. 1), after which 105 were removed as irrelevant, leaving 107 publications for full-text review. Among these publications, 38 were not investigating the first-pass meconium or had to be excluded due to not having performed NGS analysis, leaving 69 which explored the following factors influencing the meconium microbiota: prenatal factors (*n* = 16), immediate perinatal factors, including exposure to antibiotics at birth and delivery mode (*n* = 11), both pre- and perinatal factors (*n* = 24) or other factors (*n* = 18) (Supplementary Information 3). The mean sample size in the publications included was 107 meconium samples with a median of 61 and a range from 8 to 950 (Supplementary Information 3). Of the publications 10 performed a sequencing analysis beyond the standard 16 S of hypervariable regions, including full-length 16 S gene sequencing and whole-genome shotgun sequencing, four used qPCR for quantitative analyses and 13 included a predicted metabolic pathway analysis (Supplementary Information 3).

Due to the importance of contamination control, we reviewed the use of technical controls in the included publications. Reporting of using technical controls varied: more than half of the publications (*n* = 37) did not mention the use of negative or positive controls, and out of the 32 that did report either negative or positive controls (including mock communities, fecal samples, or DNA extractions of singular bacteria), 16 failed to mention the number of negative controls used and four the number of positive controls (Supplementary Information 3). In six studies, a control sample, usually a diaper sample, had already been taken during sample collection to examine possible contaminations (Supplementary Information 3).

Bacterial DNA was found in meconium in all except one included publication. The most commonly studied prenatal factors were gestational age (*n* = 18) and maternal pregnancy-related health issues (*n* = 13), while the most common immediate perinatal factor was delivery mode (*n* = 33) and the most common newborn-related factor was the size of the newborn at birth (*n* = 11) (Supplementary Information 3). Altogether 24 publications explored the effects of both prenatal and immediate perinatal factors, and 13 of these used bi or multivariate analyses to assess the effects of pre- and perinatal factors on the composition of the meconium microbiota (Supplementary Information 3). The publications were highly heterogeneous regarding what factors were studied and whether their health outcomes were investigated. Overall, the results in all the publications were mixed and inconclusive regarding the effects of pre- and perinatal factors on meconium microbiota. Altogether 17 of 33 studies, analyzing the effect of delivery mode on the meconium microbiota and possible subsequent health, yielded no significant differences. Of the ones that did, vaginally delivered infants’ meconium tended to be enriched with *Escherichia-Shigella, Lactobacillus, Bacteroides,* and *Bifidobacterium*, whereas infants’ born via C-section meconium had more *Staphylococcus* and *Corynebacterium*. In some studies, C-section delivery and the resulting meconium microbiota was investigated as a risk factor for future health conditions. Generally, maternal pregnancy-related health issues and newborn-related outcomes were found to be associated with an altered meconium microbiota.

### Population characteristics in the cohort study

A total of 393 mother-neonate pairs were enrolled in the cohort study, 186 involving children born vaginally and 207 via C-section (Table 1).

**Table 1:** Population characteristics of the 393 mother-newborn pairs, including prenatal, immediate perinatal and newborn factors.

Population characteristics	Cohort size *n* = 393
Prenatal factors	
Maternal age (years) mean (SD)	30.8 (5.5)
Maternal weight at the start of pregnancy (kg) mean (SD)^a^	69 (15)
Maternal weight at the end of pregnancy (kg) mean (SD)^b^	83 (16)
Maternal asthma *n* (%)	31 (7.6)
Maternal allergy *n* (%)	122 (31.0)
Gestational diabetes *n* (%)^c^	102 (26.0)
Smoking during pregnancy *n* (%)	38 (9.7)
* Streptococcus agalactiae-*positive^d^	77 (19.6)
Antibiotics during pregnancy *n* (%)	98 (24.9)
Mean h/week (SD) spent in a forest^e^	3.1 (4.2)
Number of siblings, mean (SD)	1.6 (2.4)
Fish consumption/week, mean (SD)^f^	1.0 (0.7)
Meat consumption/week, mean (SD)^g^	5.3 (2.5)
Immediate perinatal factors	
Delivery mode, vaginal delivery *n* (%)	186 (47.3)
Antibiotics during delivery *n* (%)	246 (62.6)
Newborn factors	
Sex (girl) (%)	188 (47.8)
Gestational age (weeks) mean (SD)	39.2 (1.6)
Birth weight (grams) mean (SD)	3500 (570)
Birth length (cm) mean (SD)^h^	50 (2.2)
Head circumference (cm) mean (SD)^i^	35 (1.7)
Apgar 1 min mean (SD)	8.7 (0.9)
Apgar 5 min mean (SD)	9.1 (0.7)
Apgar 15 min mean (SD)	9.3 (0.6)
Postnatal antibiotics n (%)^j^	17 (4.3)
Meconium sampling time h (SD)^k^	9.3 (8.0)

^a^Weight at the start of pregnancy not available for 12 mothers.

^b^Weight at the end of pregnancy not available for 8 mothers.

^c^Defined by abnormal oral glucose tolerance values.

^d^*Streptococcus agalactiae* screening result not reported for 86 mothers.

^e^Weekly time spent in a forest not available for 30 mothers.

^f^Fish consumption not available for 17 mothers.

^g^Meat consumption not available for 15 mothers.

^h^Birth length not available for 8 newborns.

^i^Head circumference not available for 9 newborns.

^j^8 children received a combination of benzyl penicillin and tobramycin, 8 received benzyl penicillin alone and 1 received a combination of cefuroxime and tobramycin.

^k^Sampling time not available for 76 meconium samples.

### Immediate perinatal factors influencing the microbiota of the meconium

**Fig. 2:**
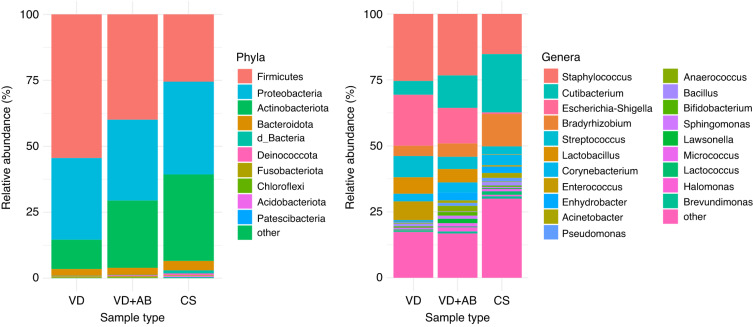
Bacterial composition of the meconium samples, by delivery mode and the use of intrapartum antibiotics. VD Vaginal delivery, no intrapartum antibiotics, VD + AB: Vaginal delivery, intrapartum antibiotics, CS: C-section. The 10 most abundant phyla and 20 most abundant genera are listed. The remaining taxa in both plots are assigned to the category “other”.

We first analyzed how the mode of delivery and exposure to antibiotics during delivery affected the composition of the meconium microbiota. The 341 samples obtained after preprocessing were divided into three groups: vaginal delivery samples without intrapartum antibiotics (VD, *n* = 118), vaginal delivery samples with intrapartum antibiotics (VD + AB, *n* = 39) and C-section delivery samples (CS, *n* = 184). Comparison of the proportions of the various phyla and genera in the meconium samples showed Firmicutes (VD: 54%, VD + AB: 40%, CS: 26%), Proteobacteria (VD: 31%, VD + AB: 31%, CS: 35%) and Actinobacteriota (VD: 11%, VD + AB: 25%, CS: 33%) to be the most abundant phyla and *Staphylococcus* the most abundant genus in the vaginal delivery samples (VD: 25%, VD + AB: 23%, CS: 15%) followed by *Escherichia-Shigella* (VD: 19%, VD + AB: 13%, CS: 0.62%) and *Streptococcus* (VD: 8.1%, VD + AB: 4.7%, CS: 3%) (Fig. 2 and Supplementary Information 4). *Cutibacterium* was the most abundant genus in the CS group (VD: 5.2%, VD + AB: 12%, CS: 22%), (Fig. 2 and Supplementary Information 4).

**Fig. 3:**
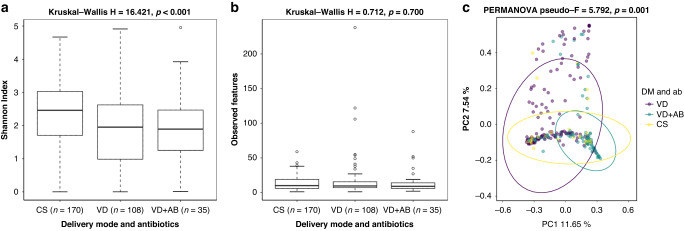
Alpha and beta diversity of meconium samples. **a** Shannon Index, **b** observed features, and **c** Bray–Curtis Dissimilarity of the meconium samples grouped according to delivery mode and exposure to intrapartum antibiotics. VD Vaginal delivery, no intrapartum antibiotics, VD + AB: Vaginal delivery, intrapartum antibiotics. CS C-section.

We then analyzed the effect of delivery mode and exposure to intrapartum antibiotics on the alpha and beta diversities of the meconium samples. After rarefying, we had 108 samples from the VD group, 35 from the VD + AB group and 170 from the CS group for the diversity analyses. The Shannon Index showed significant differences between the VD and CS groups (*p* < 0.001) and between the VD + AB and CS groups (*p* = 0.016), while the samples from the infants born via C-section were slightly more diverse in their bacterial composition than the vaginal delivery samples in general (Fig. 3a and Supplementary Information 5). The mean number (SD) of observed features in the samples was low in all the groups, however: 17 (27) in VD, 14 (17) in VD + AB, and 13 (9.4) in CS, yielding no significant differences between the groups (Fig. 3b and Supplementary Information 5). In the beta diversity analysis, the meconium samples differed between the VD, VD + AB and CS groups (*p* = 0.001), and significant differences were found between all the sample groups in the pairwise comparisons of the beta diversity analyses (Fig. 3c and Supplementary Information 5).

ANCOM analysis showed that both delivery mode and exposure to intrapartum antibiotics contributed to the differences in abundance between the samples. Several such differences in phyla and genera were found when comparing the VD and CS groups and in the phyla Actinobacteriota and Firmicutes between the VD and VD + AB groups, while no differentially abundant phyla or genera could be identified between the VD + AB and CS group (Table 2).

**Table 2:** Differentially abundant features identified when using ANCOM to compare the sample groups.

Pairwise comparison	Phyla	W	Genera	W
VD vs. VD + AB	Actinobacteriota	14	NA	NA
VD vs. CS	Actinobacteriota	20	*Cutibacterium*	430
	Firmicutes	20	*Escherichia-Shigella*	429
			*Lactobacillus*	426
			*Enterococcus*	421
			*Bradyrhizobium*	417
VD + AB vs. CS	NA	NA	NA	NA

Differences in abundance were measured at the phylum and genus levels. W: Number of times the zero hypothesis was rejected.

### Prenatal factors influencing the microbiota of the meconium

In addition, we performed a univariate analysis of the whole cohort using alpha and beta diversities in the various prenatal factors, including environmental and maternal factors and the newborn size (Supplementary Information 6). The presence of furry pets at home during pregnancy was associated with the number of observed features (*p* = 0.024) but the alpha and beta diversity analyses showed no significant differences in any other prenatal factors (Supplementary Information 6). We also performed an ANCOM analysis on the same prenatal factors and found small differences in some of them (Supplementary Information 7). The mother’s weight and weight gain during pregnancy affected the abundance of *Pelomonas*, maternal use of medicines, mostly iron supplements, painkillers, thyroid hormones and blood pressure medicine, and likewise smoking, affected the abundance of *Acidovorax*, and the consumption of fish and meat during pregnancy affected the abundance of *Acidiphilium* (Supplementary Information 7). Finally, the newborn’s size at birth influenced the abundance of the genera *Rahnella1* and *Bryocella* (Supplementary Information 7).

### Multivariate linear mixed model

We used a multivariate linear mixed model to compare the relative abundances of 10 taxa, the prevalence of which ranged from 32 to 303 zero counts in the samples studied, in the presence of 5 prenatal and 2 immediate perinatal factors (Table 3). When adjusted for these factors in the model, this analysis showed that the phylum Actinobacteriota was significantly more abundant in infants exposed to intrapartum antibiotics than in those not exposed, while the abundance of Firmicutes and *Escherichia-Shigella* was significantly greater in the vaginal delivery samples and *Cutibacterium* was in the C-section samples (Supplementary Information 8 and 9). Furthermore, *Enterococcus* was significantly more abundant in the meconium samples when there were no older siblings in the household during pregnancy (Supplementary Information 8 and 9). The rest of the comparisons yielded no statistically significant results (Supplementary Information 8 and 9).

**Table 3:** Pre-and perinatal factors used in the multivariate mixed model and the outcome variables, including 4 phyla, 6 genera and 2 alpha diversity metrics analyzed here.

Factors studied		Outcome variables	
Immediate perinatal factors	Prenatal factors	Taxa in the model	Alpha diversity metrics
Delivery mode	Maternal age	Actinobacteriota*	Shannon Index
Intrapartum antibiotics	Maternal forest exposure	Bacteroidota	Chao1
	Maternal weight gain	Firmicutes*	
	Presence of furry pets	Proteobacteria	
	Presence of older siblings	Cutibacterium*	
		*Enterococcus*#	
		Escherichia-Shigella*	
		*Lactobacillus*	
		*Staphylococcus*	
		*Streptococcus*	

Statistically significant taxa based on immediate perinatal factors have been marked with an asterisk (*), and based on prenatal factors with a hashtag (#). Detailed information can be seen in Supplementary Information 8 and 9.

### PICRUSt2 analysis of the meconium microbiota

**Fig. 4:**
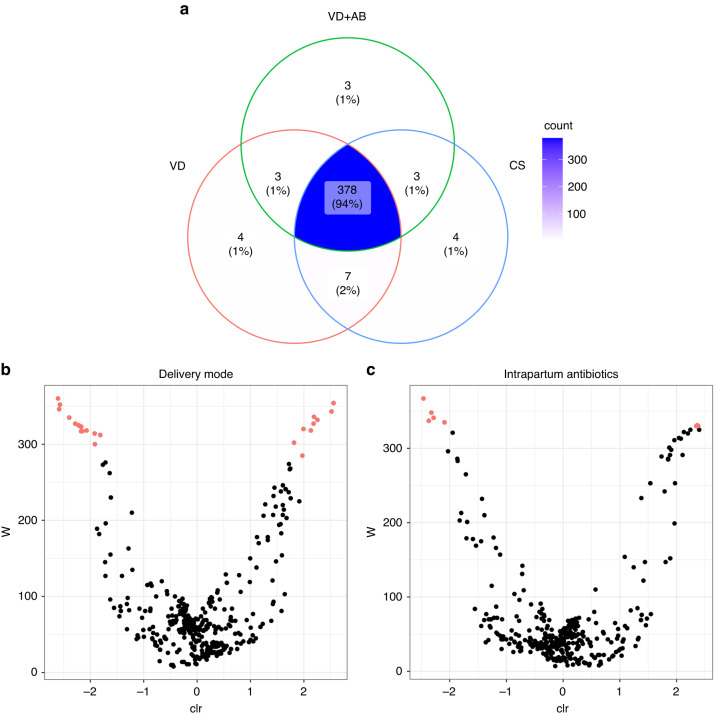
PICRUSt2 results of meconium samples based on the mode of delivery and exposure to intrapartum antibiotics. **a** A Venn diagram of predicted metabolic pathways shared between the sample groups. **b** Volcano plot of the abundances of the predicted metabolic pathways that differed according to the delivery mode. **c** Volcano plot of the predicted metabolic pathway abundances that differed according to intrapartum antibiotic exposure. Pathways that differ in abundance are colored red. The pathway labels were removed from the plots due to overlaps.

A predicted metabolic pathway analysis performed using the 16 S data for the newborns in our cohort identified a total of 401 predicted pathways, 378 (94%) of which were shared between all three sample groups (Fig. 4a). There were a few pathways that were unique to one group: 4 to VD, 3 to VD + AB and 4 to CS (Fig. 4a). Using ANCOM, we found pathways that differed in abundance depending on both the delivery mode (25 pathways) and exposure to intrapartum antibiotics (8 pathways; see Fig. 4b, c and Supplementary Information 10). The pathways that differed according to the use of intrapartum antibiotics were mainly those involved in fatty acid biosynthesis, the degradation of various metabolites and aerobic respiration, while those that different according to the delivery mode included those responsible for metabolite degradation, biosynthesis of membrane structures, biosynthesis of metabolites and amino acids, aerobic respiration, fatty acid biosynthesis and the urea cycle (Supplementary Information 10).

### Sample classification using machine learning to predict the delivery mode

**Fig. 5:**
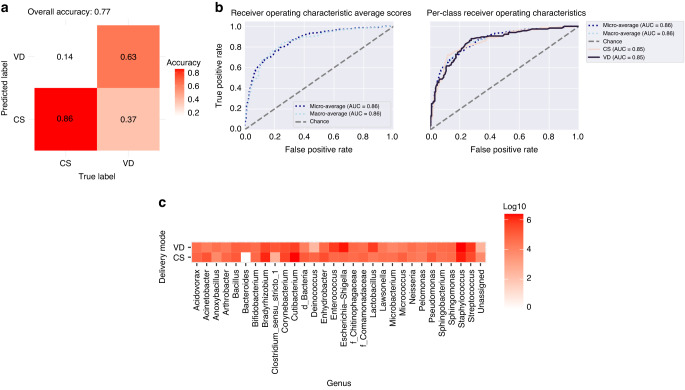
Random forest classification of the samples according to delivery mode. **a** The proportions of the samples that were correctly and incorrectly assigned to the given mode. “True label” (*x*-axis) means the true delivery mode of the samples and “Predicted label” (*y*-axis) the delivery mode that the classifier assigned to the samples. **b** Receiver operating characteristics (ROC) of classifier accuracy, with the area under the curve (AUC) depicted for both the CS and VD samples, together with the micro- and macro-average AUCs. **c** The 30 most important genera for classifying samples according to delivery mode. The numbers of reads are presented as log10 scores.

Using the random forest (RF) learning method for classifying our samples, we obtained a model in which the RF classifier managed to predict the delivery mode in 77% of the samples, C-section deliveries being correctly identified more often, at an accuracy rate of 0.86 (86%), while vaginal delivery samples without intrapartum antibiotic treatment were correctly identified with 0.63 (63%) accuracy (Fig. 5a). Receiver Operating Characteristics (ROC) curves for both delivery modes had an area under curve (AUC) of 0.85 (Fig. 5b). The 30 most important genera for distinguishing between the delivery modes are listed in Fig. 5c.

## Discussion

The systematic review of the literature provided some comparative evidence for the effects of prenatal and immediate perinatal factors on the initial colonization of the gut in neonates, while the cohort study indicated that this early gut colonization was altered at birth according to the mode of delivery and exposure to intrapartum antibiotics. Furthermore, the multivariate model constructed here showed that immediate perinatal factors influenced the microbial composition of the first-pass meconium more than did prenatal factors. This finding supports the idea that the microbiota of the first stool is mainly influenced by the delivery.

The largest differences in the meconium microbiota were found here between infants born via the vaginal route without intrapartum antibiotic treatment and those born via C-section. This finding was further supported by our machine learning analysis, which was able to identify C-section samples accurately. Half of the 33 studies in our systematic review that examined the delivery mode as a contributor to meconium microbiota development^2,5,6,13–15,36–60^ found that it did indeed have an effect. This implies that if the colonization process starts *in utero* or during birth, interventions to influence the composition of the infant’s gut microbiota may need to be started early. The importance of an early intervention is emphasized by previous reports that the meconium microbiota has been found to be associated with the subsequent health of the infants, such as neonatal jaundice,^46,61,62^ NEC,^40,60,63,64^ allergies and atopy,^65,66^ disrupted infant growth,^10,53,67–71^ early-onset neonatal sepsis (EONS)^60,72,73^ and other conditions.^9,60,74–76^ One possible intervention to alter the gut microbiota may be the use of probiotics, which have been found to have a positive effect on the newborn gut.^60,77^

In clinical practice the use of intrapartum antibiotics is often linked with the delivery mode, since intrapartum antibiotics are used to prevent early-onset group B streptococcal (GBS) sepsis in vaginal deliveries usually by administering intravenous penicillin to the 20–30% of pregnant women who are colonized with GBS^78–80^ while almost all women undergoing C-section are given antibiotics to prevent surgical site infections.^80^ Thus it is difficult to determine whether the meconium microbiota is shaped by the delivery mode, the use of intrapartum antibiotics, or the combined effect of both. Studies investigating the effect of antenatal^37,39,40,49,57,58,64,69,81^ and intrapartum^13,36,45,72,82^ antibiotics on the meconium microbiota have shown variable results. In the present cohort study, intrapartum antibiotics seemed to alter the meconium microbiota development in the infants born vaginally, suggesting that maternal exposure to antibiotics during vaginal birth acts rapidly to modify early gut colonization, probably before the actual birth.

The microbial composition of the first-pass meconium appearing here to be affected mainly by immediate perinatal factors suggests that the meconium microbiota is mostly formed during delivery and not during the fetal period, although we cannot rule out the possibility that the fetus may be exposed to maternal microbiota or microbiota-derived extracellular vesicles^23^ or metabolites^83^ allowing prenatal factors to modify these contacts *in utero*. In addition, we collected extensive data on maternal living environments and lifestyles during pregnancy, such as fish and meat consumption, leisure time spent in forests, the presence of older siblings or furry pets in the home, smoking in the home and maternal weight, and established that these prenatal factors had little effect on the microbial composition of meconium. It is therefore understandable that the prenatal factors considered in our systematic review, including maternal diet,^2,37,84,85^ residential area,^37,44^ age,^37,44,49,50,53,57,86^ ethnicity,^3,49^ weight,^3,38,44,49,53,56,57,75,86^ smoking,^3,37,44,87^ pregnancy-related health issues,^36–40,47,49,54,60,71,75,85,86^ non-pregnancy-related health issues^3,37,38,44,45,49,70,76,88,89^ and education,^37,44,49^ together with environmental pollutants, particularly metals and microplastics^58,90^ and the presence of furry pets^13,45^ yielded mixed results. Another commonly studied newborn factor, which we did not analyze in our cohort study, was gestational age,^3,4,36–39,44,45,48–50,52,53,57,59,72,91–93^ which most reports have found to affect the development of the meconium microbiota. Interestingly, when Yang et al. compared the meconium microbiota of monozygotic twins with that of dizygotic ones, they concluded that the former resembled each other in terms of their gut microbiota more than did the latter,^94^ implying a possible intrauterine colonization mechanism. These results emphasize the controversial nature of gut microbiota development in newborns as a research topic and point to a need for a carefully planned and controlled study design especially in the case of a low-biomass microbiota.

PICRUSt2 analysis to predict metabolic pathways in our samples yielded significant differences in the abundances of the predicted pathways according to the delivery mode and to a lesser extent the use of intrapartum antibiotics. This suggests that the delivery mode especially affects the major metabolic pathways in meconium microbiota, which may contribute to the function of the microbiota at birth. This contradicts earlier PICRUSt findings, as one study mentions a difference in the pathways of transitional stool, but not in the meconium.^42^ Furthermore, in an earlier study where shotgun metagenomic sequencing was applied to both meconium and stool collected 1 month after birth, differences in the taxonomic and gene composition were found in later stool samples but not in meconium.^95^ Finally, studies using whole-genome shotgun sequencing and full length 16 S rRNA gene sequencing did not find significant differences in the meconium samples based on the delivery mode.^2,5^

The strength of the present study lies in the combination of a systematic review of the literature with a comparison of the impact of prenatal and immediate perinatal factors on the microbiota of the first stool in a large cohort using a multivariate model, together with the use of a large dataset of environmental factors and extensive analyses of their effects on the meconium microbiota. To our knowledge, this is overall the largest cohort study characterizing the meconium microbiota and the environmental factors affecting it to be published to date. We used various methods, including machine learning and predicted metabolic pathway analysis. Since reagent and other laboratory contaminations are common in sequencing studies^17^ it is crucial in microbiota research to be able to identify such contamination, so that extraneous microbes will not be mistaken for true microbiota findings. We had a large number of negative controls to enable decontamination processes to be introduced during the analysis. This is especially important for low-biomass microbiota research, such as that involving the meconium.

The study had certain limitations, however. We were unable to perform sequencing of the full 16 S gene due to the low biomass of the meconium samples. Although sequencing of the full gene might have improved the accuracy of the bacterial taxonomy to the species level, sequencing of the V3-V4 region of the gene is still common in microbiota studies, even with the possible primer biases.^96^ Furthermore, we did not include negative controls such as diaper samples to cover possible contamination during sample collection. The genus *Cutibacterium* is a skin commensal and may be a contamination rather than a true colonizer in the microbiota of the meconium. In an earlier study, we analyzed the diapers of newborns alongside their meconium samples and found that the diapers did not contaminate the meconium microbiota.^13^ Finally, we were limited in our data analysis in many cases by the small group sizes or by missing data, which may have affected the results.

In conclusion, the systematic review showed that there is not yet a consensus on when the meconium microbiota develops or what factors affect its development. The minor effect of prenatal factors on meconium microbiota development in comparison to the observed effect of immediate perinatal factors, including the mode of delivery and exposure to intrapartum antibiotics, suggests that the initial human gut microbiota is mostly influenced by the delivery. The delivery likely shapes the infant gut microbiota development after birth.

### Supplementary Information

Supplementary_information1,2,4,5,7,8,9,10

Supplementary_information_3

Supplementary_information_6

## Data Availability

Raw sequences were submitted to Genbank with the Bioproject number PRJNA905086.
