# Supplementary material for: Investigating prenatal and perinatal factors on meconium microbiota: a systematic review and cohort study
Source: Pediatr Res. 2023 Aug 17;95(1):135–45. doi: 10.1038/s41390-023-02783-z (PMC10798900; doi:10.1038/s41390-023-02783-z)
Supplement: Supplementary file 1 — Supplementary_information1,2,4,5,7,8,9,10 [file 41390_2023_2783_MOESM1_ESM.pdf]

**Supplementary information 1.** Query search terms, dates, and results for each database.

| **Search parameter** | **Items found/included** |
|---|---|
| **Pubmed Query search on 01-02-2022** | |
| ((("meconium"[MeSH Terms] OR "meconium"[All Fields] OR "meconiums"[All Fields]) AND ("microbiome s"[All Fields] OR "microbiomic"[All Fields] OR "microbiomics"[All Fields] OR "microbiota"[MeSH Terms] OR "microbiota"[All Fields] OR "microbiome"[All Fields] OR "microbiomes"[All Fields])) OR (("meconium"[MeSH Terms] OR "meconium"[All Fields] OR "meconiums"[All Fields]) AND ("microbiota"[MeSH Terms] OR "microbiota"[All Fields] OR "microbiotas"[All Fields] OR "microbiota s"[All Fields] OR "microbiotae"[All Fields]))) AND ((humans[Filter]) AND (english[Filter])) | 121 |
| Language filter | English |
| Population filter | Human |
| **Scopus Query search on 01-02-2022** | |
| TITLE-ABS-KEY(meconium microbiome OR meconium microbiota) AND ( LIMIT-TO ( LANGUAGE,"English" ) ) AND ( LIMIT-TO ( EXACTKEYWORD,"Human" ) ) | 110 |
| Language filter | English |
| Population filter | Human |
| Duplicates | 83 |
| **Web of Science Query search on 02-02-2022** | |
| (ALL=(meconium microbiome OR meconium microbiota)) AND (LA==("ENGLISH")) | 178 |
| Language filter | English |
| Duplicates | 114 |
| Total screened after duplicate removal | 212 |
| Total articles after exclusion | 69 |

**Supplementary information 2.** A list of TruSeq adapter sequences used as truncated overhangs during the 2-step PCR protocols first round of PCR

| Adapter name | Adapter sequence |
|---|---|
| F1 | ATCTACACTCTTTCCCTACACGACGCTCTTCCGATCT |
| F2 | ATCTACACTCTTTCCCTACACGACGCTCTTCCGATCTgt |
| F3 | ATCTACACTCTTTCCCTACACGACGCTCTTCCGATCTagag |
| F4 | ATCTACACTCTTTCCCTACACGACGCTCTTCCGATCTtagtgt |
| R1 | GTGACTGGAGTTCAGACGTGTGCTCTTCCGATCT |
| R2 | GTGACTGGAGTTCAGACGTGTGCTCTTCCGATCTa |
| R3 | GTGACTGGAGTTCAGACGTGTGCTCTTCCGATCTtct |
| R4 | GTGACTGGAGTTCAGACGTGTGCTCTTCCGATCTctgagtg |

**Supplementary information 4.** Relative frequencies of bacterial composition of meconium samples based on the delivery mode and the usage of intrapartum antibiotics. Top ten most frequent taxa are shown, and the rest are collapsed in the “other” category.

| **Vaginal delivery** | | | | **Vaginal delivery with intrapartum antibiotics** | | | |
|---|---|---|---|---|---|---|---|
| **Phyla (%)** | | **Genera (%)** | | **Phyla (%)** | | **Genera (%)** | |
| Firmicutes | 54 | *Staphylococcus* | 25 | Firmicutes | 40 | *Staphylococcus* | 23 |
| Proteobacteria | 31 | *Escherichia-Shigella* | 19 | Proteobacteria | 31 | *Escherichia-Shigella* | 13 |
| Actinobacteriota | 11 | *Streptococcus* | 8.1 | Actinobacteriota | 25 | *Cutibacterium* | 12 |
| Bacteroidota | 2.5 | *Enterococcus* | 7.1 | Bacteroidota | 2.7 | *Lactobacillus* | 5.1 |
| Fusobacteriota | 0.57 | *Lactobacillus* | 6.2 | Deinococcota | 0.38 | *Bradyrhizobium* | 5.1 |
| d__Bacteria | 0.14 | *Cutibacterium* | 5.2 | Fusobacteriota | 0.36 | *Streptococcus* | 4.7 |
| Verrucomicrobiota | 0.11 | *Bradyrhizobium* | 3.9 | Chloroflexi | 0.32 | *Corynebacterium* | 3.8 |
| Deinococcota | 0.096 | *Corynebacterium* | 2.9 | d__Bacteria | 0.18 | *Enhydrobacter* | 3.0 |
| Patescibacteria | 0.019 | *Clostridium_sensu_stricto_1* | 1.7 | Fibrobacterota | 0.017 | *Anaerococcus* | 2.1 |
| Unassigned | 0.0035 | *Bacillus* | 0.95 | Planctomycetota | 0.010 | *Lawsonella* | 1.7 |
| other | 0.00025 | other | 19 | other | 0.016 | other | 26 |

| **C-section delivery** | | | |
|---|---|---|---|
| **Phyla (%)** | | **Genera (%)** | |
| Proteobacteria | 35 | *Cutibacterium* | 22 |
| Actinobacteriota | 33 | *Staphylococcus* | 15 |
| Firmicutes | 26 | *Bradyrhizobium* | 12 |
| Bacteroidota | 3.6 | *Corynebacterium* | 4.0 |
| d__Bacteria | 1.2 | *Streptococcus* | 3.0 |
| Deinococcota | 0.70 | *Enhydrobacter* | 2.4 |
| Acidobacteriota | 0.37 | *Acinetobacter* | 1.9 |
| Fusobacteriota | 0.20 | *Pseudomonas* | 1.4 |
| Patescibacteria | 0.16 | *Lactococcus* | 1.3 |
| Chloroflexi | 0.11 | *Bacillus* | 1.3 |
| other | 0.27 | other | 35 |

**Supplementary information 5.** Kruskal-Wallis H pairwise comparisons between each sample group of alpha diversity metrices Shannon Index and observed features as well as beta diversity using PCoA with Bray-Curtis dissimilarity. Each group's sample size n is included. The table includes Kruskal-Wallis H and the corrected p-value using Benjamini-Hochberg correction method as well as p-value of the PERMANOVA test. Significant values have been marked in bold.

| Group comparisons | | Shannon Index | | Observed features | | Bray-Curtis dissimilarity |
|---|---|---|---|---|---|---|
| | | H | p-value | H | p-value | PERMANOVA p-value |
| VD (n=109) | VD+AB (n=35) | 0.034 | 0.855 | 0.795 | 0.373 | **0.001** |
| VD (n=109) | CS (n=171) | **14.148** | **<0.001** | 0.027 | 0.870 | **0.001** |
| VD+AB (n=35) | CS (n=171) | **5.850** | **0.016** | 0.499 | 0.480 | **0.003** |

**Supplementary information 7.** ANCOM results of the newborn, maternal, and environmental factors. Differentially abundant features have been analyzed in Phyla and Genera level.

| **Prenatal (maternal) factors** | **Phyla** | **W** | **Genera** | **W** |
|---|---|---|---|---|
| Age of the mother during pregnancy (<30 or ≥30 years) | no | no | no | no |
| Weight of the mother at the beginning of the pregnancy (<65 or ≥65 kg) | no | no | *Pelomonas* | 411 |
| Weight of the mother at the end of the pregnancy (<80 or ≥80 kg) | no | no | *Pelomonas* | 414 |
| Weight gain of the mother during pregnancy (<15 or ≥15 kg) | no | no | Pelomonas | 423 |
| Maternal GDM | Acidobacteriota | 7 | c__Bacilli | 321 |
| Maternal lactic acid bacteria usage during pregnancy | no | no | *Corynebacteriaceae*<br>f__Beijerinckiaceae | 155<br>115 |
| Maternal antibiotics during pregnancy | no | no | no | no |
| Maternal medicine during pregnancy | no | no | *Acidovorax* | 445 |
| Maternal smoking during pregnancy | no | no | *Acidovorax* | 444 |
| Maternal fish consumption (<1, 1, or >1 per week) | Acidobacteriota | 11 | *Acidiphilium* | 347 |
| Maternal meat consumption (<5, 5-6, or 6> per week) | Acidobacteriota | 14 | *Acidiphilium* | 363 |
| **Prenatal (environmental) factors** | **Phyla** | **W** | **Genera** | **W** |
| Forest exposure of the mother during pregnancy (<2 or ≥2 hours) | no | no | no | no |
| Owning a summer cottage | no | no | no | no |
| Presence of furry pets in the household during pregnancy | no | no | no | no |
| Presence of older siblings in the household during pregnancy | no | no | no | no |
| **Postnatal factors** | **Phyla** | **W** | **Genera** | **W** |
| Sex of the newborn | no | no | no | no |
| Newborn's weight (<3500 or ≥3500 g) | no | no | no | no |
| Newborn's height (<50 or ≥50 cm) | Acidobacteriota | 14 | *Rahnella1*<br>*Bryocella* | 394<br>354 |
| Head circumference of the newborn at birth (<35 or ≥35 cm) | Acidobacteriota | 13 | *Rahnella1*<br>*Bryocella* | 364<br>327 |

**Supplementary information 8.** Regression analysis using linear mixed model on the most abundant phyla. We analyzed seven different delivery and maternal factors. Factors with p-values of ≤0.05 have been marked in bold.

| Phyla | Factor | Mean Difference | p-value | 95% CI | |
|---|---|---|---|---|---|
| | | | | Lower | Upper |
| Actinobacteriota | **intrapartum ab (no, yes)** | **-0.087** | **0.031** | **-0.116** | **-0.008** |
| | delivery mode (vd, cs) | -0.062 | 0.118 | -0.141 | 0.016 |
| | forest exposure h (<2, ≥2) | -0.024 | 0.776 | -0.090 | 0.042 |
| | older siblings (no, yes) | -0.002 | 0.956 | -0.059 | 0.056 |
| | furry pets (no, yes) | 0.007 | 0.795 | -0.046 | 0.059 |
| | mom weight gain kg (<15, ≥15) | -0.021 | 0.809 | -0.084 | 0.042 |
| | mom age y (<30, ≥30) | -0.003 | 0.912 | -0.056 | 0.050 |
| Firmicutes | intrapartum ab (no, yes) | 0.087 | 0.094 | -0.015 | 0.189 |
| | **delivery mode (vd, cs)** | **0.153** | **0.003** | **0.052** | **0.225** |
| | forest exposure h (<2, ≥2) | 0.053 | 0.364 | -0.033 | 0.138 |
| | older siblings (no, yes) | 0.009 | 0.809 | -0.065 | 0.084 |
| | furry pets (no, yes) | 0.024 | 0.494 | -0.044 | 0.092 |
| | mom weight gain kg (<15, ≥15) | 0.019 | 0.926 | -0.062 | 0.100 |
| | mom age y (<30, ≥30) | 0.016 | 0.649 | -0.053 | 0.085 |
| Proteobacteria | intrapartum ab (no, yes) | 0.012 | 0.823 | -0.090 | 0.113 |
| | delivery mode (vd, cs) | -0.078 | 0.129 | -0.178 | 0.023 |
| | forest exposure h (<2, ≥2) | -0.035 | 0.690 | -0.120 | 0.050 |
| | older siblings (no, yes) | -0.010 | 0.783 | -0.084 | 0.063 |
| | furry pets (no, yes) | -0.023 | 0.508 | -0.090 | 0.045 |
| | mom weight gain kg (<15, ≥15) | 0.023 | 0.872 | -0.058 | 0.103 |
| | mom age y (<30, ≥30) | -0.013 | 0.718 | -0.081 | 0.056 |
| Bacteroidota | intrapartum ab (no, yes) | -0.006 | 0.751 | -0.044 | 0.032 |
| | delivery mode (vd, cs) | -0.002 | 0.921 | -0.040 | 0.036 |
| | forest exposure h (<2, ≥2) | 0.004 | 0.986 | -0.028 | 0.036 |
| | older siblings (no, yes) | 0.003 | 0.853 | -0.025 | 0.030 |
| | furry pets (no, yes) | -0.008 | 0.534 | -0.033 | 0.017 |
| | mom weight gain kg (<15, ≥15) | -0.014 | 0.586 | -0.045 | 0.016 |
| | mom age y (<30, ≥30) | 0.000 | 0.974 | -0.026 | 0.025 |

**Supplementary information 9.** Regression analysis using linear mixed model on the most interesting genera. We analyzed seven different delivery and maternal factors. Factors with p-values of ≤0.05 have been marked in bold.

| Genera | Factor | Mean Difference | p-value | 95% CI | |
|---|---|---|---|---|---|
| | | | | Lower | Upper |
| *Lactobacillus* | intrapartum ab (no, yes) | 0.011 | 0.579 | -0.027 | 0.048 |
| | delivery mode (vd, cs) | 0.035 | 0.071 | -0.003 | 0.072 |
| | forest exposure h (<2, ≥2) | 0.009 | 0.856 | -0.022 | 0.041 |
| | older siblings (no, yes) | 0.018 | 0.205 | -0.010 | 0.046 |
| | furry pets (no, yes) | 0.003 | 0.818 | -0.022 | 0.028 |
| | mom weight gain kg (<15, ≥15) | 0.014 | 0.596 | -0.016 | 0.045 |
| | mom age y (<30, ≥30) | 0.005 | 0.682 | -0.021 | 0.031 |
| *Streptococcus* | intrapartum ab (no, yes) | 0.011 | 0.615 | -0.033 | 0.055 |
| | delivery mode (vd, cs) | 0.019 | 0.397 | -0.025 | 0.063 |
| | forest exposure h (<2, ≥2) | 0.003 | 0.995 | -0.034 | 0.040 |
| | older siblings (no, yes) | 0.018 | 0.267 | -0.014 | 0.051 |
| | furry pets (no, yes) | 0.015 | 0.312 | -0.014 | 0.045 |
| | mom weight gain kg (<15, ≥15) | -0.003 | 0.994 | -0.039 | 0.032 |
| | mom age y (<30, ≥30) | 0.013 | 0.402 | -0.017 | 0.043 |
| *Escherichia-Shigella* | intrapartum ab (no, yes) | 0.035 | 0.213 | -0.020 | 0.090 |
| | **delivery mode (vd, cs)** | **0.065** | **0.020** | **0.010** | **0.120** |
| | forest exposure h (<2, ≥2) | 0.004 | 0.996 | -0.043 | 0.050 |
| | older siblings (no, yes) | -0.002 | 0.914 | -0.043 | 0.038 |
| | furry pets (no, yes) | -0.011 | 0.575 | -0.048 | 0.027 |
| | mom weight gain kg (<15, ≥15) | -0.006 | 0.985 | -0.050 | 0.039 |
| | mom age y (<30, ≥30) | -0.002 | 0.930 | -0.040 | 0.036 |
| *Staphylococcus* | intrapartum ab (no, yes) | 0.008 | 0.833 | -0.064 | 0.079 |
| | delivery mode (vd, cs) | 0.041 | 0.255 | -0.030 | 0.113 |
| | forest exposure h (<2, ≥2) | 0.022 | 0.772 | -0.039 | 0.082 |
| | older siblings (no, yes) | -0.045 | 0.096 | -0.098 | 0.008 |
| | furry pets (no, yes) | -0.009 | 0.706 | -0.058 | 0.039 |
| | mom weight gain kg (<15, ≥15) | 0.015 | 0.889 | -0.042 | 0.073 |
| | mom age y (<30, ≥30) | 0.024 | 0.330 | -0.025 | 0.074 |
| *Enterococcus* | intrapartum ab (no, yes) | 0.031 | 0.084 | -0.004 | 0.065 |
| | delivery mode (vd, cs) | 0.003 | 0.845 | -0.031 | 0.038 |
| | forest exposure h (<2, ≥2) | 0.018 | 0.350 | -0.011 | 0.047 |
| | **older siblings (no, yes)** | **0.034** | **0.010** | **0.008** | **0.059** |
| | furry pets (no, yes) | -0.015 | 0.192 | -0.039 | 0.008 |
| | mom weight gain kg (<15, ≥15) | -0.006 | 0.931 | -0.034 | 0.022 |
| | mom age y (<30, ≥30) | -0.022 | 0.074 | -0.045 | 0.002 |
| *Cutibacterium* | intrapartum ab (no, yes) | -0.053 | 0.096 | -0.115 | 0.009 |
| | **delivery mode (vd, cs)** | **-0.074** | **0.020** | **-0.135** | **-0.012** |
| | forest exposure h (<2, ≥2) | 0.014 | 0.884 | -0.038 | 0.067 |
| | older siblings (no, yes) | -0.009 | 0.707 | -0.055 | 0.037 |
| | furry pets (no, yes) | 0.015 | 0.479 | -0.027 | 0.057 |
| | mom weight gain kg (<15, ≥15) | -0.036 | 0.236 | -0.086 | 0.014 |
| | mom age y (<30, ≥30) | -0.001 | 0.958 | -0.044 | 0.042 |

**Supplementary information 10.** Differentially abundant predicted metabolic pathways based on the delivery mode and the usage of intrapartum antibiotics in vaginal delivery samples. Metacyc.org was used to find detailed pathway names.

| **Delivery mode** | | |
|---|---|---|
| Pathway name | Detailed pathway name | W |
| FUC-RHAMCAT-PWY | superpathway of fucose and rhamnose degradation | 360 |
| PWY-7094 | fatty acid salvage | 354 |
| GLYCOL-GLYOXDEG-PWY | superpathway of glycol metabolism and degradation | 352 |
| GLCMANNANAUT-PWY | superpathway of N-acetylglucosamine, N-acetylmannosamine and N-acetylneuraminate degradation | 346 |
| PROTOCATECHUATE-ORTHO-CLEAVAGE-PWY | protocatechuate degradation II (ortho-cleavage pathway) | 343 |
| PWY-5920 | superpathway of heme b biosynthesis from glycine | 336 |
| PWY0-41 | allantoin degradation IV (anaerobic) | 335 |
| LEU-DEG2-PWY | L-leucine degradation I | 332 |
| PWY-6629 | superpathway of L-tryptophan biosynthesis | 327 |
| PWY-7431 | aromatic biogenic amine degradation (bacteria) | 327 |
| PWY0-1277 | 3-phenylpropanoate and 3-(3-hydroxyphenyl) propanoate degradation | 325 |
| HCAMHPDEG-PWY | 3-phenylpropanoate and 3-(3-hydroxyphenyl)propanoate degradation to 2-hydroxypentadienoate | 324 |
| PWY-6690 | cinnamate and 3-hydroxycinnamate degradation to 2-hydroxypentadienoate | 324 |
| PWY-7446 | sulfoquinovose degradation I | 323 |
| P108-PWY | pyruvate fermentation to propanoate I | 320 |
| ECASYN-PWY | enterobacterial common antigen biosynthesis | 318 |
| PWY-4984 | urea cycle | 318 |
| P161-PWY | acetylene degradation (anaerobic) | 317 |
| PWY-7315 | dTDP-N-acetylthomosamine biosynthesis | 317 |
| ORNDEG-PWY | superpathway of ornithine degradation | 314 |
| ARGDEG-PWY | superpathway of L-arginine, putrescine, and 4-aminobutanoate degradation | 312 |
| ORNARGDEG-PWY | superpathway of L-arginine and L-ornithine degradation | 312 |
| PWY-3781 | aerobic respiration I (cytochrome c) | 302 |
| KDO-NAGLIPASYN-PWY | superpathway of $(Kdo)_2$-lipid A biosynthesis | 300 |
| PWYG-321 | mycolate biosynthesis | 285 |
| **Intrapartum antibiotics** | | |
| Pathway name | Detailed pathway name | W |
| PWY-7094 | fatty acid salvage | 367 |
| P108-PWY | pyruvate fermentation to propanoate I | 348 |
| PWY-7431 | aromatic biogenic amine degradation (bacteria) | 341 |
| PROTOCATECHUATE-ORTHO-CLEAVAGE-PWY | protocatechuate degradation II (ortho-cleavage pathway) | 337 |
| PWY-3781 | aerobic respiration I (cytochrome c) | 335 |
| GLYCOL-GLYOXDEG-PWY | superpathway of glycol metabolism and degradation | 331 |
| FUC-RHAMCAT-PWY | superpathway of fucose and rhamnose degradation | 330 |
| PWY0-41 | allantoin degradation IV (anaerobic) | 330 |
